# Trajectory of plasma syndecan‐1 and heparan sulphate during major surgery: A retrospective observational study

**DOI:** 10.1111/aas.14150

**Published:** 2022-09-27

**Authors:** Laurence Weinberg, Fumitaka Yanase, Shervin Tosif, Bernhard Riedel, Rinaldo Bellomo, Robert G. Hahn

**Affiliations:** ^1^ Department of Anaesthesia Austin Hospital Melbourne Australia; ^2^ Department of Critical Care The University of Melbourne Melbourne Australia; ^3^ Department of Intensive Care Austin Hospital Melbourne Australia; ^4^ Australian and New Zealand Intensive Care Research Centre (ANZIC‐RC), School of Public Health and Preventative Medicine Monash University Melbourne Australia; ^5^ Department of Anaesthesia, Perioperative and Pain Medicine, Peter MacCallum Cancer Centre and the Sir Peter MacCallum Department of Oncology University of Melbourne Melbourne Australia; ^6^ Karolinska Institute at Danderyd's Hospital (KIDS) Stockholm Sweden; ^7^ Department of Research Södertälje Hospital Södertälje Sweden

**Keywords:** anaesthesia, fluids, glycocalyx, inflammation, surgery

## Abstract

**Background:**

Surgical trauma‐induced inflammation during major surgery may disrupt endothelial integrity and affect plasma concentrations of glycocalyx constituents, such as syndecan‐1 and heparan sulphate. To date, no studies have focused on their perioperative temporal changes.

**Methods:**

As part of a trial, we obtained plasma and urine specimens sampled during the perioperative period in 72 patients undergoing major abdominal surgery. The plasma concentration of syndecan‐1 and heparan sulphate was measured on five occasions, from baseline to the second postoperative day. Plasma and urinary creatinine and urinary syndecan‐1 concentrations were measured before surgery and on the first postoperative morning.

**Results:**

We observed three different temporal patterns of plasma syndecan‐1 concentration. Group 1 ‘low’ (64% of patients) showed only minor changes from baseline despite a median heparan sulphate increase of 67% (*p* < .005). Group 2 ‘increase’ (21% of patients) showed a marked increase in median plasma syndecan‐1 from 27 μg/L to 118 μg/L during the first postoperative day (*p* < .001) with a substantial (+670%; *p* < .005) increase in median plasma heparan sulphate from 279 to 2196 μg/L. Group 3 ‘high’ (14% of patients) showed a constant elevation of plasma syndecan‐1 to >100 μg/L, but low heparan sulphate levels. The plasma C‐reactive protein concentration did not differ across the three groups and 90% of colon surgeries occurred in Group 1. Treatment with dexamethasone was similar across the three groups. Surgical blood loss, duration of surgery and liver resection were greatest in Group 2.

**Conclusion:**

Changes in syndecan‐1 and heparan sulphate after surgery appear to show three different patterns, with the greatest increases in those patients with greater blood loss, more liver surgery and longer operations. These observations suggest that increases in syndecan‐1 and heparan sulphate reflect the degree of surgical injury.

**Editorial Comment**

Perioperative changes of the glycocalyx constituents syndecan‐1 and heparan sulphate were studied up to the second postoperative day in patients undergoing major abdominal surgery. Greater intraoperative insult from blood loss, length or type of surgery was reflected in greater syndecan‐1 and heparan sulphate plasma concentrations. These findings may guide further research on the impact of surgery on endothelial function and by extension fluid resuscitation and haemostasis.

## 1 INTRODUCTION

The glycocalyx is a thin 0.2–0.5 μm layer of glycosylated proteins that covers the luminal side of the endothelium. Moreover, the glycocalyx is hypothesised to govern local capillary permeability, vasodilatation, coagulation and inflammation.^1^, ^2^, ^3^ The integrity of the glycocalyx can be disrupted by ischaemia‐reperfusion injury, inflammation due to surgical trauma and aggressive fluid overload with release of B‐natriuretic peptide—which may lead to the appearance of excess quantities of glycocalyx fragments in the peripheral blood.^4^, ^5^, ^6^ Shedding of the glycocalyx during surgery is therefore important as it may lead to dysregulation of inflammation and vascular permeability resulting in the development of postoperative morbidity as indicated by an increased rate of severe complications, length of stay, anticipated admissions to the intensive care unit and hospital readmissions post‐surgery.

**FIGURE 1:**
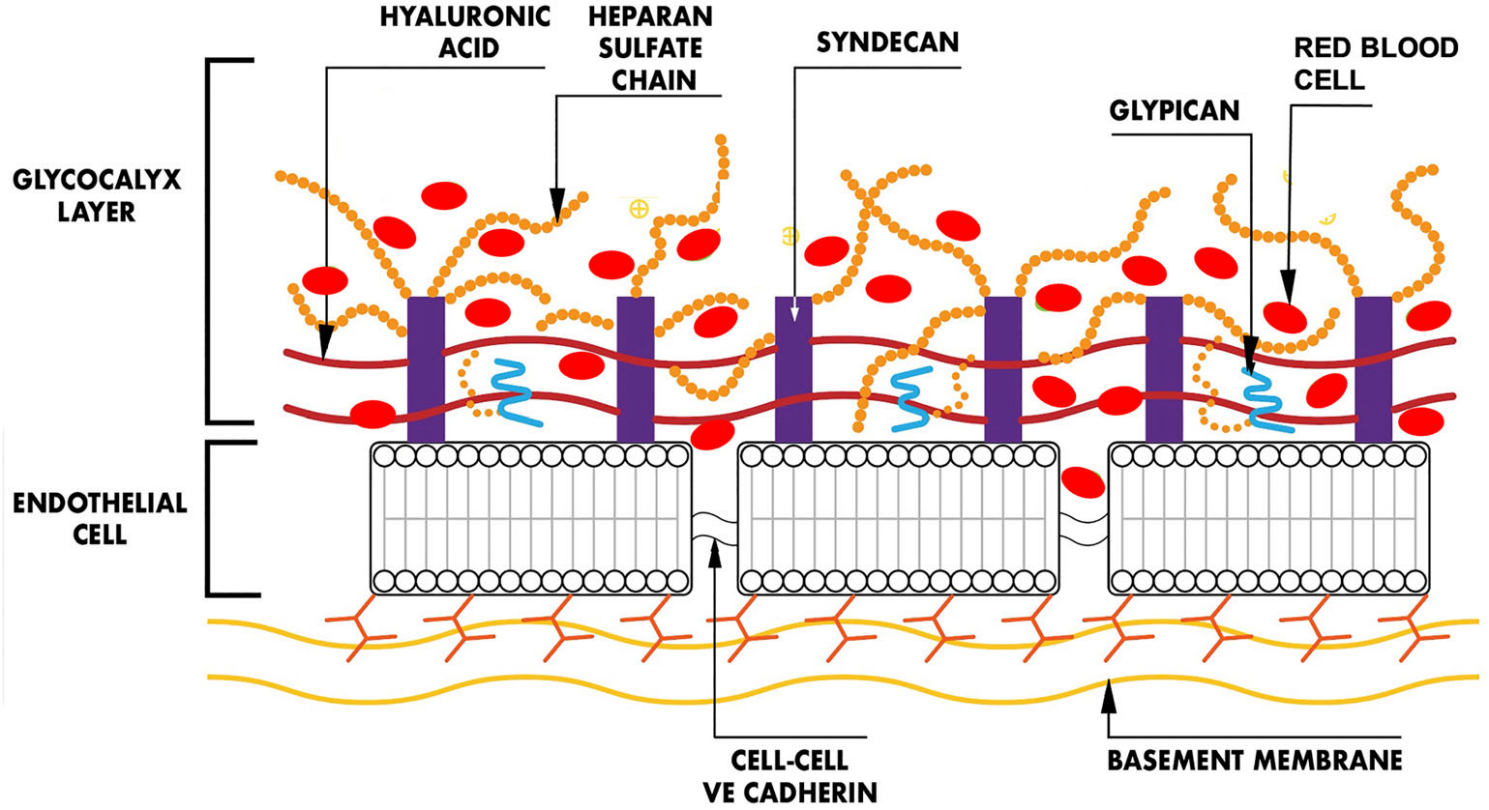
The endothelial glycocalyx depicting an inflamed state with red blood cells penetrating the glycocalyx and within the cell junctions

Syndecan‐1 and heparan sulphate are two biomarkers commonly used to assess such endothelial damage (Figure 1). Syndecan‐1 is a 32 kD proteoglycan that is coded by the sdc‐1 gene. This molecule serves as a membrane protein but has an extracellular component to which heparan sulphate and chondroitin sulphate side chains attached. The disruption of the glycocalyx during surgery, inflammation and/or ischaemia‐reperfusion injury, with release into the peripheral circulation is termed ‘shedding’.^7^, ^8^ Plasma concentrations are often doubled, tripled or quadrupled^8^; however, even greater elevations of syndecan‐1 have been reported after cardiac surgery and burn injury.^6^, ^9^ Anaesthesia drugs and infusion of fluids may cause moderate degrees of shedding.^10^, ^11^, ^12^ However, researchers typically report various pointwise measurements of shedding products in plasma without providing a temporal, longitudinal overview of the changes, or absence of changes, at the individual level.

The purpose of the present study was to assess whether there are patterns of ‘shedding’ of glycocalyx molecules during the perioperative period of major surgery. For this purpose, we undertook a post hoc analysis of data from a prospective study where we examined the effect of dexamethasone and 20% albumin solution on preserving endothelial integrity in the perioperative setting.^13^ The hypothesis of this post hoc study was that distinct patterns could be discerned, which would be associated with patient factors, operative characteristics and/or postoperative outcomes.

## 2 METHODS

### 2.1 Permissions

This is a secondary report of a clinical pilot study of 72 adult patients (≥18 years old) undergoing open pancreas, colorectal and liver resection surgeries between July 2018 and January 2019.^13^ The protocol was approved by the local Ethics Committee approval (number: 17/Austin/397) and was registered with the Australian New Zealand Clinical Trial Registry before any patient was recruited (ACTRN: 12618000361202). Data were collected at two Australian hospitals and one New Zealand hospital. Written informed consent was obtained from all participants before they were enrolled in the trial. Ethics approval/exemption was obtained for this post hoc analysis.

### 2.2 Purpose

The primary aim of the pilot study was to explore whether the plasma concentrations of two endothelial shedding products—syndecan‐1 and heparan sulphate—measured from before to 2 days after major abdominal surgery—could be altered by two strategies (albumin and dexamethasone) that may be endothelial protective. General management of the patients was described in the primary publication.^13^ Specifically, we explored the impact of a cortisone analogue (dexamethasone, as an anti‐inflammatory agent that prevents cytokine‐related injury to the glycocalyx) and albumin (which through negative anionic binding to the glycocalyx may be protective) on endothelial integrity. For this purpose, patients were randomised to receive dexamethasone (16 mg) and 100 ml of 20% albumin solution intravenously over 30 min prior to skin incision. The control group was given 100 ml of a physiological balanced salt solution (i.e., Plasmalyte or Hartmann's solution) over 30 min prior to skin incision. Both groups received the same balanced salt solution for maintenance and fluid resuscitation.

### 2.3 Blood chemistry

Blood samples were collected at five intervals during the perioperative period: before induction of anaesthesia, at the end of surgery, during the night after surgery and on the mornings of postoperative days 1 and 2. Blood from all five intervals was used to measure the plasma concentration of syndecan‐1 (ELISA, Diaclone, France; coefficient of variation of 6.2%) samples from before surgery and on the first postoperative morning for plasma concentration of heparan sulphate (ELISA, Amsbio, Abingdon, UK; with a coefficient of variation of <10%). These ELISAs were performed on batched samples at a single research laboratory facility. Blood from the same timepoints was analysed to measure the plasma concentrations of creatinine and C‐reactive protein at the central clinical chemistry laboratory at each study site.

### 2.4 Urine analysis

An indwelling catheter was placed in the bladder immediately upon induction of general anaesthesia (baseline), and a mid‐portion urine sample was sent for measurement of albumin, creatinine and syndecan‐1 concentrations. Urine was collected via an indwelling catheter in the bladder during the first postoperative night and up to the morning of postoperative day 1. Urine flow rate was recorded, and samples were sent for analysis of albumin, creatinine and syndecan‐1 concentrations. The urine flow prior to initiation of surgery was inferred from the urinary creatinine concentration, assuming a constant excretion. The renal clearance of syndecan‐1 was calculated as the product of the urine flow rate and the ratio between the urine and plasma concentrations of syndecan‐1.

### 2.5 Statistics

The data were reported as the median (25th–75th percentile limits) due to the frequent occurrence of skewed distributions. Selected comparisons were made using the Mann–Whitney and chi‐square tests. The Wilcoxon matched pair test was used to compare measurements at different times. *p* < .05 was considered statistically significant. The data were analysed using the SPSS Statistics predictive analytic software (IBM SPSS Statistics for Windows, Version 28.0, Armonk, NY).

## 3 RESULTS

**FIGURE 2:**
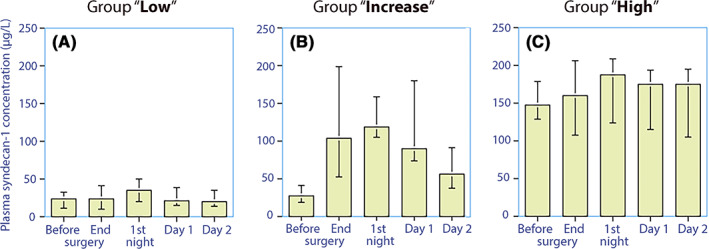
(A–C) Three patterns of plasma syndecan‐1 concentrations in the perioperative. Data presented as median (25th–75th centiles)

Data were missing from two patients; therefore, the final analysis was based on 70 subjects. Postoperative urine values were missing for 16 patients. Visual inspection of the temporal trajectories of plasma syndecan‐1 suggested the presence of three different patterns (Figure 2).

### 3.1 Group 1 ‘low’

**FIGURE 3:**
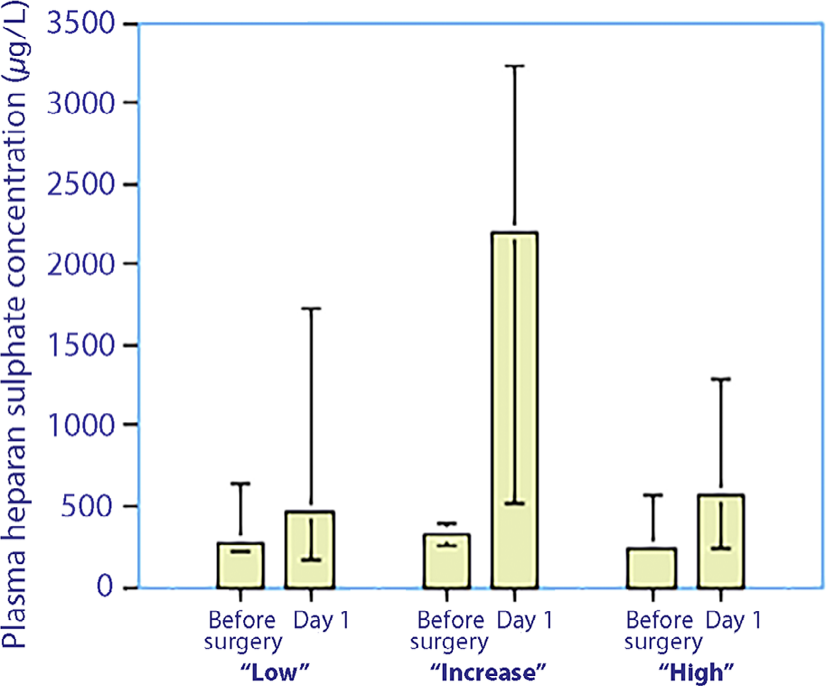
Plasma concentrations of heparan sulphate measured just before initiation of surgery and on the first postoperative morning, based on the classification of the temporal trajectory of plasma syndecan‐1 as low, increasing or high. Data presented as median (25th–75th centiles)

**FIGURE 4:**
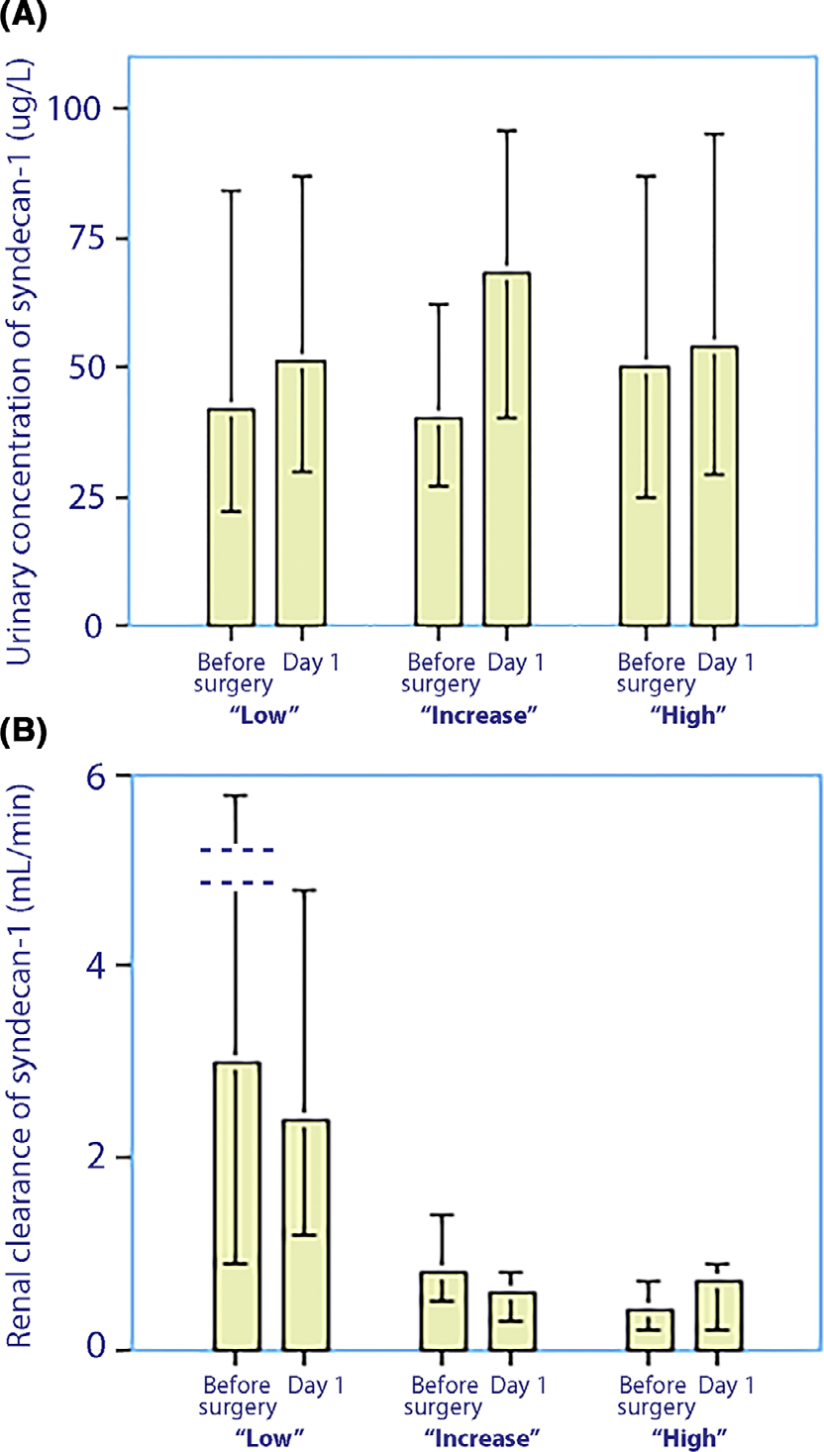
(A) Urinary concentrations of syndecan‐1 and (B) renal clearance of syndecan‐1 measured just before initiation of surgery and on the first postoperative morning, depending on classification of the trajectory of plasma syndecan‐1 as low, increasing or high. Data presented as median (25th–75th centiles)

Forty‐five patients (64% of the study population) had a low syndecan‐1 concentration in plasma throughout the perioperative period, with all values similar to baseline values for subjects who have not undergone surgery.^14^, ^15^, ^16^ Thus, median plasma syndecan‐1 increased from 21 (12–33) μg/L to 24 (11–42) μg/L up to the end of surgery (*p* < .01; Figure 2A). In contrast, plasma heparan sulphate increased from 279 (219–645) μg/L to 466 (167–1723) μg/L (+67% increase from baseline, *p* < .005; Figure 3). The urinary syndecan‐1 concentration was unchanged (Figure 4A). The renal clearance of syndecan‐1 was estimated as 3.0 (0.9–8.6) ml/min before the surgery and estimated as 2.4 (1.2–4.8) ml/min on the first postoperative morning (*p* = .08; Figure 4B).

### 3.2 Group 2 ‘increase’

Fifteen patients (21%) had an increase in median plasma syndecan‐1 from 27 (19–41) μg/L to 103 (52–98) μg/L during the surgery (*p* < .003) and the highest median value at 118 (105–158) during the first postoperative night (Figure 2B). In contrast, plasma heparan sulphate increased from a median of 329 (248–387) μg/L to 2196 (515–3222) μg/L during the surgery (+670% increase from baseline, *p* < .005; Figure 3).

Median urinary syndecan‐1 concentration changed from 40 (27–62) μg/L to 68 (40–96) μg/L (*p* = .09; Figure 4A). Median renal clearance of syndecan‐1 was 0.8 (0.5–1.4) ml/min before surgery and 0.6 (0.3–0.8) ml/min after surgery (*p* < .79; Figure 4B). These clearances were significantly lower than in Group 1 (*p* < .001 for postoperative data).

### 3.3 Group 3 ‘high’

In contrast to the above two groups, Syndecan‐1 concentrations were consistently elevated in this group. The median plasma concentration was 147 (128–178) μg/L before surgery and 159 (107–206) μg/L at the end of surgery (*p* < .84) and reached the highest value of 187 (123–208) during the first postoperative night (Figure 2C). Plasma heparan sulphate increased from 244 (197–569) μg/L to 566 (231–1280) μg/L during surgery (+230% increase from baseline, *p* < .049; Figure 3). The urinary syndecan‐1 concentration did not change (*p* = .99; Figure 4A). The renal clearance of syndecan‐1 was 0.4 (0.2–0.7) ml/min before surgery and 0.7 (0.2–0.9) ml/min on the first postoperative morning (Figure 4B). These clearances were significantly lower than those in the ‘Group low’ (*p* < .013 for preoperative value and *p* < .003 for the postoperative value).

### 3.4 Demographic differences

Table 1 shows the demographic and biochemical data for the three groups. The most apparent between‐group difference was that 90% of the colon surgeries occurred in Group ‘low’. The operating time was shorter for Group ‘low’ than for Group ‘high’; even though the mean durations of all surgeries were similar.

**TABLE 1:** Demographics and selected data on the surgical procedures

Variable	Group ‘low’	Group ‘increase’	Group ‘high’	*p* value
Number (*N*)	45	15	10	
Age (years)	62 (53–70)	66 (59–73)	57 (48–70)	.08
Gender (females)	24 (53%)	7 (53%)	3 (30%)	.39
ASA Class I/II/III	2/22/21	0/4/11	0/4/6	.56
Body mass index (kg/m^2^)	27.1 (24.1–29.7)	24.0 (21.1–29.2)	29.4 (21.9–35.3)	.34
Smoker (*N*, %)	8 (16%)	2 (13%)	0 (0%)	.35
Diabetes (*N*, %)	6 (13%)	4 (27%)	4 (40%)	.12
Metastasis (*N*, %)	5 (11%)	5 (33%)	3 (30%)	.18
Colon/pancreas/liver surgery (*N*)	21/16/8	0/5/10	2/3/5	.002
%	47/36/17	0/33/67	20/30/50	
Operating time (h)	5.3 (3.9–6.3)	7.8 (6.0–10.7)	5.0 (4.3–6.1)	.022
Laparoscopy (*N*, %)	10 (22%)	0 (0%)	1 (10%)	.10
Surgical blood loss (ml)	100 (50–300)	325 (100–800)	300 (50–500)	.001
Received dexamethasone (*N*, %)	26 (58%)	5 (33%)	5 (50%)	.26

*Note*: Continuous data are presented as median and 25th–75th percentiles. Incidence data were compared using contingency table analysis and continuous data using the Kruskal–Wallis test.

### 3.5 Biochemical differences

Selected biochemical data are shown in Table 2. The plasma concentration of C‐reactive protein increased to the same degree in all three groups. Treatment with dexamethasone was associated with a lower postoperative C‐reactive protein concentration (48 [29–78] vs. 84 [45–133] mg/L, *p* < .003) but not with a lower plasma syndecan‐1 levels at any time (*p* range: .62–.97). Only a marginal increase was noted in the urine albumin/creatinine ratio (*p* < .08). Plasma creatinine increased by >50% in five patients in Group ‘increase’ (33%), while only two similar elevations (4%) occurred in Group ‘low’ and none in Group ‘high’ (*p* < .001 and *p* < .02 when comparing these two groups with the ‘low’ group). Group ‘increase’ had the lowest creatinine clearance on the first postoperative morning.

**TABLE 2:** Indices of inflammation and kidney function before surgery started (baseline) and on the first postoperative morning

Variable	Group ‘low’	Group ‘increase’	Group ‘high’	*p* value
C‐reactive protein				
Before (mg/L)	3 (2–7)	4 (1–13)	3 (1–12)	.76
After (mg/L)	61 (4–91)	57 (30–96)	58 (35–113)	.85
Serum creatinine				
Before (μmol/L)	66 (57–78)	70 (61–74)	68 (57–80)	.92
After/before (ratio)	1.02 (0.90–1.16)	1.14 (0.88–1.62)	0.92 (0.83–1.13)	.29
Urine creatinine				
Before (mmol/L)	7.0 (3.9–13.5)	11.2 (5.2–14.2)	9.5 (4.9–14.2)	.77
After (mmol/L)	9.5 (6.9–12.8)	8.4 (3.9–10.8)	8.8 (7.1–9.6)	.46
Urine flow rate				
After (ml/min)	1.0 (0.7–1.7)	0.7 (0.5–1.1)	1.5 (1.0–1.7)	.13
Urine albumin/creatinine				
Before (mg/mmol)	1.8 (1.0–5.4)	2.4 (0.8–6.5)	1.6 (1.0–3.6)	.78
After (mg/mmol)	2.8 (1.4–6.6)	5.0 (1.3–9.3)	2.9 (1.8–5.7)	.95
Creatinine clearance				
Before (ml/min)^a^	136 (95–180)	105 (76–124)	195 (145–239)	.08
After (ml/min)	131 (83–186)	74 (55–104)	219 (154–237)	.004

*Note*: Data are the median and 25th–75th percentiles. Incidence data were compared using contingency table analysis and continuous data using the Kruskal–Wallis test.

^a^ Postoperative urine flow was measured, but the preoperative urine flow was estimated from urinary creatinine.

## 4 DISCUSSION

### 4.1 Key findings

We conducted a detailed assessment of changes in syndecan‐1 levels and heparan sulphate levels as well as changes in syndecan‐1 clearance and other relevant biochemical variables in the perioperative period to test the hypothesis that specific patterns of postoperative release would be observed and that such patterns would be associated with demographic and/or operative features. We found that three patterns could be identified with a group of ‘low’ change, another with marked increase and a third with persistently high levels beginning at baseline. Moreover, we found that patients with low levels were characterised by having colonic surgery, while those with marked increases had more blood loss, longer surgery and underwent open liver resection surgery. Finally, we found that change in syndecan clearance was minor and that there was no association between changes in glycocalyx shedding markers and C‐reactive protein.

### 4.2 Relationship to previous studies

Previous studies have shown that the plasma concentration of syndecan‐1 1 day after major surgery was almost the same as the concentration in volunteers^15^ and that abdominal hysterectomy elicited only a postoperative elevation of heparan sulphate.^16^ Measurement of the plasma concentrations of glycocalyx constituents is a common method for assessing endothelial injury and a more pragmatic and less cumbersome method compared to measuring the thickness of the glycocalyx using specialised imaging.^17^, ^18^ Further, elevated glycocalyx degradation products in the plasma are associated with poor prognosis in trauma^19^, ^20^ and sepsis^18^, ^21^, ^22^, ^23^ The most crucial consequence during surgery is that shedding increases the permeability of capillaries to macromolecules such as albumin, thereby reducing the colloid oncotic pressure and intravascular plasma volume.^24^, ^25^ However, the sensitivity or specificity of these plasma concentrations as indicators of changed capillary permeability is still unknown.

In the present study, we observed distinct differences in the temporal trajectory of the plasma concentration of syndecan‐1, despite similar protein C responses to the surgery. In Group ‘low‘, the heparan sulphate concentrations in plasma almost doubled while syndecan‐1 concentrations only rose marginally. This similar pattern has previously been reported,^16^ with one plausible explanation being that syndecan‐1 is more resistant to injury than the side branches. Isolated shedding of syndecan‐1 seems unlikely due to the ultrastructural arrangement of the glycocalyx.^8^ Syndecan‐1 is a core protein with the edge embedded into the endothelial cell membrane with up to three heparan sulphate molecules attached as side branches on its endoluminal side. Syndecan‐1 shedding increases greatly when the heparan sulphate chains have been enzymatically degraded from the core protein.^26^ This is consistent with our findings of syndecan‐1 concentrations in the Group ‘low’, and with our findings of the greater increase of heparan sulphate accompanying the marked elevation of syndecan‐1 in Group ‘increase’. Hence, we postulate that shedding occurred in Group ‘low’ but was of a lesser intensity than in Group ‘increase’, which likely incurred a significant endothelial injury. Other factors contributing to this difference in shedding may include longer operating time, greater bleeding and a greater incidence of liver surgery.

Interestingly, in the Group ‘high’ the plasma heparan sulphate concentrations were similar to those found in Group ‘low’, while the syndecan‐1 levels were constantly very high, and markedly elevated even before surgery began. One hypothesis to explain this unexpected finding is that the syndecan‐1 in this group did not originate in the endothelium but instead from a body organ. Glycosaminoglycans may also be expressed at multiple sites, such as in the liver.^13^

### 4.3 Kidney function

The urinary concentration of shedding products is typically in the same range as in the plasma. The molecules are small (30 kD), which makes urinary excretion a likely main route of elimination. In the present study, the urinary concentration was even twice as high in Group ‘low’, which indicates that the entire plasma pool of syndecan‐1 would have been excreted from the start of surgery until the first postoperative morning.

The urinary excretion of heparan sulphate is known to increase with the plasma concentration, whereas the urinary excretion of syndecan‐1 varies with the creatinine clearance and the urine flow.^27^ Hence, only the renal elimination of heparan sulphate becomes more effective when the plasma concentration is high, although some dependency on kidney function has also been reported.^28^

Prerequisites for effective elimination of syndecan‐1 are good kidney function and high urine flow, which may both be acutely depressed by anaesthesia and surgery. The excretion is *not* strongly dependent on the plasma concentration; therefore, the relationship between the renal clearance and plasma concentration of syndecan‐1 has an inverse character, which can be discerned by comparing Figure 2 with Figure 4B.^27^, ^28^ The fact that the same amount of syndecan‐1 is excreted even when the plasma concentration becomes very high makes the kidneys unable to effectively correct high concentrations.

Several indications of impaired kidney function are apparent in Group ‘increase’. The urine flow was lower, as was the creatinine clearance, than in the other groups. More patients also had a postoperative increase in plasma creatinine that surpassed 50% of baseline, thereby fulfilling a criterion for Stage I Acute Kidney Injury.^29^

### 4.4 Implications

The present study demonstrates that significant variations in syndecan‐1 plasma concentrations can be expected in patients undergoing major abdominal surgery, despite their virtually identical C‐reactive protein concentrations. Given that heparan sulphate has been reported to occur in all animal tissues, our study provides further evidence that as a proteoglycan it may regulate biological activities in colon, liver and pancreatic tissues. Further, our findings suggest that extensive surgical trauma seems to be required to release syndecan‐1 fragments into circulation.

However, further research is needed to better understand whether the major cell membrane heparan sulphate proteoglycans—syndecan‐1 is primarily responsible for maintaining the integrity of the glycocalyx in the context of inflammation due to surgical trauma, hence also being a possible ‘therapeutic target’ in patients undergoing major surgery (similar to its potential role as a target for cancer immunotherapy^30^, ^31^), or whether other minor forms of membrane heparan sulphate proteoglycans, for example, beta‐glycans,^32^ play more of a role in protecting the glycocalyx in the context of major liver, colon and pancreatic surgeries. Finally, whilst the relation between endothelial glycocalyx shedding and increased vascular permeability is generally well accepted, recent studies report that degradation of the endothelial glycocalyx in an animal model of non‐haemorrhagic shock results in the vascular barrier permeability remaining intact, challenging the concept that the glycocalyx barrier is a significant contributor to vascular barrier permeability.^33^

### 4.5 Strengths and limitations

The strengths of the present study include that the data were obtained under carefully standardised conditions. The series is also sufficiently large to allow the identification of subgroups with different syndecan‐1 responses. However, the report is a post hoc analysis from a randomised clinical trial of the effect of dexamethasone and 20% albumin on endothelial integrity during major surgery,^13^ where our exploratory findings suggested that this strategy may associate with a reduction in postoperative complications. The analysis was motivated by visual inspection of the data, which suggested huge between‐patient variabilities in syndecan‐1 before surgery and as a response to surgery. The randomised study was not blinded. Group ‘high’ included only 10 patients. Postoperative urine was missing from 16 patients, which further reduced the power in the analyses of urine‐based variables. The calculation of the renal clearance of syndecan‐1 assumes that the urinary content originates from the plasma; however, this is not certain. Many details regarding the metabolism of syndecan‐1 are unknown; further, the possibility that syndecan‐1 in the urine is released from the kidneys cannot be excluded.

## 5 CONCLUSIONS

The temporal trajectory of syndecan‐1 varied greatly between patients during the perioperative period for major surgery. Three patterns of change were identified with only one group showing an increase in syndecan‐1, which was coupled with a substantial increase in heparan sulphate. This suggests that heparan sulphate may be a more sensitive biomarker of endothelial dysfunction in this setting. Moreover, these changes did not correlate with the plasma concentration C‐reactive protein but reflected patients undergoing longer surgery, with more blood loss and open liver resection surgeries. These findings may imply a dissociation between inflammation and tissue injury as assessed by these biomarkers and suggest the need for further investigations in larger groups of patients.

## AUTHOR CONTRIBUTIONS

**Laurence Weinberg**: methodology, data collection, literature review, supervison, and writing. **Fumitaka Yanase**: data collection, literature review and writing. **Shervin Tosif**: data collection, literature review and writing. **Bernhard Riedel**: data collection, literature review and writing. **Rinaldo Bellomo**: methodology, literature review and writing. **Robert G. Hahn**: study conceptualisation, methodology, data collection, formal analysis, writing and project adminstration.

## FUNDING INFORMATION

Open access publishing facilitated by The University of Melbourne, as part of the Wiley—The University of Melbourne agreement via the Council of Australian University Librarians.

## CONFLICT OF INTEREST

The authors declare no conflicts of interest.

## PATIENT CONSENT

All participants provided written consent.

## CLINICAL TRIAL REGISTRATION

The original study was prospectively registered with the Australian New Zealand Clinical Trial Registry (ACTRN12618000361202).

## Data Availability

The full‐deidentified dataset is available from the corresponding author on reasonable request.
